# Exploring the Shared Genetic Architectures Between Primary Open-Angle Glaucoma and Visual Pathway Regions in the Brain

**DOI:** 10.1167/iovs.66.15.11

**Published:** 2025-12-02

**Authors:** Asma M. Aman, Stuart MacGregor, Santiago Diaz-Torres, Puya Gharahkhani

**Affiliations:** 1 QIMR Berghofer, Brisbane, QLD, Australia; 2 Faculty of Health, Medicine and Behavioural Sciences, The University of Queensland, Brisbane, QLD, Australia; 3 School of Biomedical Sciences, Queensland University of Technology, Brisbane, QLD, Australia

**Keywords:** glaucoma, central visual pathways, genetic overlap, candidate causal genes, colocalization analysis

## Abstract

**Purpose:**

To investigate the genetic relationships between primary open-angle glaucoma (POAG) and major visual pathways in the brain to better understand the neurological biology of glaucoma, which may facilitate the discovery of neuroprotective drug targets.

**Methods:**

We assessed the relationship between POAG and the volumes of five visual pathway regions using genetic correlation and polygenic risk score (PRS). We further used Mendelian randomization (MR) to investigate the causal relationships. In addition, we used genome-wide association study (GWAS)–pairwise analysis to identify genomic segments with shared causal variants and summary data-based MR (SMR) to uncover potential causal genes shared between POAG and the visual brain regions.

**Results:**

We found a weak but significant genetic correlation only between POAG and optic chiasm (OC) volume (rg = −0.094, *P* = 0.009). Similarly, PRS for none of the volumes of visual pathway regions showed a significant association with POAG, except for the OC volume (odds ratio = 0.96, *P* = 0.0003). In addition, no causal relationships were found between POAG and visual brain regions. The GWAS-pairwise analyses revealed several genomic segments with shared causal variants between POAG and the five brain regions; genetic loci implicated for OC included *the* *CDKN2* gene family region. In addition, the SMR analyses identified five shared potential causal genes, including *PHETA1*, *MAPKAPK5-AS1*, and *EEF1AKMT2*.

**Conclusions:**

Our findings suggest a genetic overlap between POAG and the volumes of visual pathway regions, including shared candidate causal genes. Further investigations are required to elucidate the specific role of the shared genes in the etiology of POAG and to assess their potential as neuroprotective drug targets.

Primary open-angle glaucoma (POAG) is a progressive and highly heritable optic neuropathy that is a major cause of irreversible vision loss worldwide.^1^ It is characterized by the death of the retinal ganglion cells (RGCs), which occurs predominantly due to elevated intraocular pressure (IOP).^2^ Previous research suggested that glaucoma may share common pathways with other neurodegenerative disorders, such as Alzheimer's disease,^3^^–^^5^ indicating that POAG etiology could involve central nervous system pathways. Furthermore, genes related to optic nerve vulnerability are consistently observed in POAG analyses (e.g., *SIX6* and *CDKN2B-AS1*),^6^ and their effects tend to be more pronounced in normal-tension glaucoma (NTG),^7^ where IOP remains within the statistically normal range (IOP <21 mm Hg). Research that underlies the etiology of POAG is mainly focused on IOP, while other mechanisms are largely unexplored. Consequently, there are no current neuroprotective treatments for POAG, and all treatments rely on controlling the IOP to slow down the disease progression; however, they do not prevent the optic neuropathic process.

Observational studies have shown a relationship between POAG and some vision-related brain regions. For instance, studies have demonstrated that the function and structure of the visual cortex, including primary and secondary regions, are affected in patients with glaucoma.^8^^–^^10^ In addition, previous research has shown that the size of the optic chiasm and lateral geniculate nucleus is smaller in patients with POAG, including NTG.^11^^–^^14^

However, no comprehensive studies investigate the genetic overlap and causal relationships between POAG and visual brain regions, particularly in relation to common biological pathways. Therefore, our goal was to investigate the genetic and biological relationships between POAG and visual pathway regions from retina to the visual cortex, as illustrated in the Figure, to better understand the pathophysiology of glaucoma, which may facilitate the discovery of neuroprotective drug targets.

**Figure 1.**
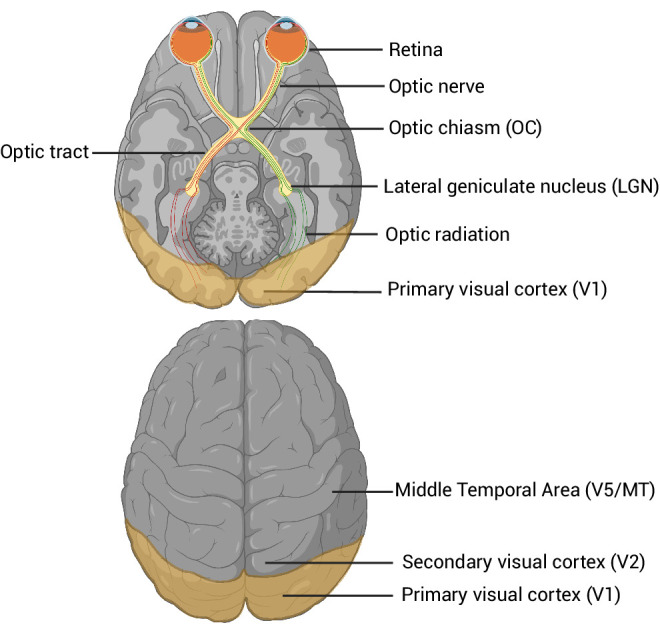
Visual pathway regions, showing the key brain structures involved in the transmission of visual information from the retina to the cortex. That includes the retina, optic nerve, optic chiasm, lateral geniculate nucleus, and primary and secondary visual cortex. Created in BioRender. Diaz S. (2025). https://BioRender.com/gdeng13.

## Methods

### Data Sources

POAG summary statistics were obtained from a multitrait genome-wide association study that combined genetic information from POAG with two key associated phenotypes, vertical cup-to-disc ratio and IOP, in 644,750 participants of European ancestry.^6^

GWASs of visual pathway regions were conducted using 41,258 European participants from the UK Biobank.^15^ The phenotypes were the volume of optic chiasm (OC; Data-Field 26530) and the mean volume from both the right and left hemispheres for the lateral geniculate nucleus (LGN; Data-Fields 26665 and 26688), primary visual cortex (V1; Data-Fields 27096 and 27138), and secondary visual cortex (V2; Data-Fields 27097 and 27139; and V5; Data-Fields 27098 and 27140). All volumes were measured using T1 structural brain magnetic resonance imaging; details on brain-imaging acquisition, processing, and quality control can be found in the UK Biobank brain-imaging articles.^16^^–^^18^

GWASs were conducted using linear mixed models to account for relatedness, with volumes transformed using the rank-based inverse normal transformation method to meet the normality assumption of the models. In addition, the GWASs were adjusted for age at imaging, sex, 10 principal components, and intracranial volume. Single-nucleotide polymorphisms (SNPs) with minor allele frequency below 0.001 and imputation quality score less than 0.3 were filtered. The analyses were performed using REGENIE software (version 2.2.4).

### Genetic Correlation

We used the linkage disequilibrium score regression (LDSC) method to estimate the genetic correlation between POAG and the volume of each visual pathway region. The LDSC is a tool used in statistical genetics that uses GWAS summary statistics instead of individual-level data and distinguishes between polygenicity and confounding.^19^^,^^20^ We used approximately 5000 healthy European participants from the UK Biobank as a linkage disequilibrium (LD) reference. The significance threshold was corrected for multiple testing using the Bonferroni method (*P* < 0.05/5 = 0.01, where 5 represents the number of visual pathway regions tested).

### Polygenic Risk Score

Polygenic risk score (PRS) is a statistical method used to summarize the effects of genetic variants on complex traits. The SBayesRC method^21^ was used to select credible SNP sets and optimize weights from the GWAS summary statistics, and then PLINK (version 2.0) was used to compute the scores. We used the GWAS summary statistics of the volume of each visual pathway region to construct the PRS to test for the association between these brain regions and the risk of developing POAG. To minimize bias, PRSs were calculated in a subset of individuals who did not overlap with the training set (i.e., those who underwent brain imaging). In addition, first-degree relatives (i.e., kinship coefficient ≥0.125) were excluded from the PRS analysis to reduce potential confounding due to relatedness. We derived the PRS using a total of 11,386 POAG cases, either self-reported or classified by the *International Classification of Diseases, 10th Revision*, and 113,724 controls from the UK Biobank. Controls were defined as individuals who did not report any eye disease. Logistic regression models were used to assess the associations, adjusting for age and sex. Similarly, we examined the association between POAG PRS and the volume of each visual pathway region. Here, the POAG PRS was constructed using summary statistics from the Million Veteran Program^22^ to avoid bias caused by sample overlap. In addition, we included visual brain volumes from 41,258 participants in the UK Biobank to derive the PRS. The volumes had a rank-based inverse normal transformation to approximate normality and facilitate comparison between traits. We fitted linear regression models to assess the associations, adjusting for age at imaging, sex, and intracranial volume as covariates. The *P* value significance threshold was adjusted using the Bonferroni method (*P* < 0.005), based on the number of visual pathway regions tested (*n* = 10).

### Causal Inference

We explored the putative causality between POAG and the volume of each visual pathway region in both directions using the Mendelian randomization (MR) method. Clumping was performed to select independent genetic instruments for the exposure, using a significance threshold of 5 × 10⁻⁸ and an LD *r*^2^ threshold of 0.001 to ensure independence between SNPs. We calculated the phenotypic variance explained by the instrumental variables included in the MR analysis for each phenotype, and the variance was ≥1%. In cases of significant heterogeneity (i.e., Cochran's *Q* test *P* < 0.05), we calculated SNP-level *Q* statistics (χ², *df* = 1) and excluded SNPs with high heterogeneity from the MR analysis.^23^ We first applied the inverse variance weighted method,^24^ followed by sensitivity analyses using MR methods, including MR-Egger regression,^25^ weighted median,^26^ and both simple and weighted mode-based estimators.^27^ Statistical significance was determined to be *P* < 0.05/10, based on the number of MR tests conducted.

### Colocalization

It is possible that local genetic overlaps can exist in the absence of genome-wide genetic correlation. We used the GWAS-pairwise (GWAS-PW) approach to identify loci shared between POAG and visual pathway regions.^28^ In this approach, the genome was divided into 1703 regions, and the posterior probability of association (PPA) was computed for each region against four hypotheses: (1) the region contains the causal genetic variant exclusively for POAG, (2) the region contains the causal genetic variant exclusively for the visual pathway region, (3) the shared region contains the common causal genetic variant for POAG and the visual pathway region, and (4) the shared region contains different causal genetic variants for POAG and the visual pathway region. The PPA threshold of 0.8 for the third model was used to select the significant shared regions.

### Identifying Overlapping Causal Genes

The multi-SNP summary data-based Mendelian randomization (SMR) method was used to test the causality between gene expression and the GWAS analysis for each phenotype,^29^^,^^30^ where we used the European UK Biobank reference LD panel. Blood expression quantitative trait loci (eQTL) data were used as genetic instruments for gene expression obtained from the eQTLGen Consortium,^31^ which is the largest eQTL database to date, with 31,684 participants. In addition, we used eQTL obtained from postmortem retinal tissues,^32^ but this data set has limited power (*n* = 453).

We assessed genes located in the shared genomic regions identified by GWAS-PW, adjusting the significance threshold for multiple testing using the Bonferroni method (*P* < 0.05/number of genes in the regions identified by GWAS-PW). The heterogeneity in dependent instruments (HEIDI) test was then used to distinguish between pleiotropy and linkage,^29^ where genes with a HEIDI *P* value more than 0.05 were considered pleiotropic genes. Afterward, we extracted and reported the overlapping significant genes between POAG and the visual pathway regions. These genes were then used to identify potential drug repurposing candidates through the Open Targets platform (https://platform.opentargets.org/).

### Expression of Prioritized Genes in Brain and Retinal Tissues

To validate the biological relevance of the prioritized genes, we retrieved their gene expression in the retinal and occipital cortex, as a proxy for the visual brain regions, from the Human Protein Atlas (HPA) platform (https://www.proteinatlas.org/),^33^ which integrates transcriptomics, proteomics, and antibody-based profiling data. Gene expressions in the occipital cortex were derived from the HPA project,^34^ while RNA expression in the retina was obtained from the Genotype-Tissue Expression v8 project,^32^^,^^35^ incorporated in the HPA platform. In addition, we used data from the Functional Annotation of Mammalian Genomes 5 (FANTOM5) project^36^ based on the cap analysis of gene expression (CAGE) data set^37^ to complement transcriptomics results with information on transcript start sites. Gene expression levels were quantified using the normalized transcripts per million (nTPM) metric, while FANTOM5 CAGE data were quantified by scaled tags per million, reflecting promoter activity. Genes were considered biologically relevant if they showed detectable expression (nTPM >1) in the occipital cortex or the retina.

Protein expression patterns were also collected from the HPA platform, where the protein expression was based on immunohistochemical staining of human tissues across cell types. Protein expression levels were qualitatively categorized by the HPA as not detected, low, medium, or high based on staining patterns.

### Expression of Prioritized Genes in Single-Cell Transcriptomics

To determine the expression in brain and eye cell types, we used publicly available single-cell transcriptomics data sets for the visual cortex^38^; glaucoma-related eye parts, including the retina^39^; and the trabecular meshwork and ciliary body.^40^ The retina, trabecular meshwork, and ciliary body were profiled using single-cell RNA sequencing, whereas the visual cortex was profiled using single-nucleus RNA sequencing, which may capture a different spectrum of transcripts and cell types. The retina data set contained 10 major classes, including photoreceptors, RGCs, and support cells such as microglia and astrocytes. The trabecular meshwork and ciliary body captured 15 distinct cell types. In addition, the visual cortex consisted of 18 cell types across three major groups: excitatory neuronal cells, inhibitory neuronal cells, and nonneuronal cells such as microglial cells and astrocytes of the cerebral cortex. Notably, nonneuronal cells represented only ∼7% of the total cells and were therefore more difficult to detect. Quality control of transcriptomics data was conducted using Seurat (v5.1.0). Cells were retained if they expressed between 2000 and 6000 genes (nFeature), had an nCount ≤50,000, and exhibited lower than 5% mitochondrial gene expression. In addition, genes were filtered to retain only those expressed in at least 0.1% of the cells to reduce noise and remove uninformative features. After filtering, 79,072 cells and 25,629 genes were retained for the retina, 125,188 cells and 24,505 genes for the trabecular meshwork and ciliary body, and 42,826 cells and 18,432 genes for the visual cortex. The data were then normalized for downstream analysis.

## Results

### Genetic Correlation

A weak but significant genetic correlation was found between POAG and OC volume (rg = −0.094, *P* = 0.009), indicating a slight inverse genetic relationship. However, there was no other significant genetic correlation between POAG and the other visual pathway regions. Genetic correlation results are shown in Table 1.

**Table 1.** LDSC Results for Genetic Correlations Between POAG and Volumes of Visual Pathway Regions

Core Trait	Trait	Estimate	SE	*P* Value*
POAG	OC	−0.094	0.036	0.009
POAG	LGN	−0.015	0.030	0.617
POAG	V1	−0.010	0.026	0.693
POAG	V2	0.006	0.028	0.835
POAG	V5	−0.028	0.030	0.356

Regions analyzed include the OC, LGN, primary visual cortex (V1), and secondary visual cortex (V2 and V5).

* A *P* value <0.01 is considered statistically significant after Bonferroni correction for five tests.

### PRS

All PRSs showed no statistically significant result except for the OC volume (odds ratio = 0.96, *P* = 3 × 10^−4^); complete logistic regression results for PRSs of visual brain regions adjusted for age and sex are presented in Table 2. In addition, linear regression results for POAG PRS are provided in Supplementary Table S1. Although the OC PRS was significantly associated with POAG, it did not provide additional predictive value compared to the age and sex model alone (area under the curve [AUC] for age and sex model: 0.701, AUC for PRS with age and sex model: 0.702).

**Table 2.** Association Between Standardized Polygenic Risk Scores for the Volumes of Visual Pathway Regions and the Risk of Developing POAG

Trait	Estimate	OR	SE	CI Lower	CI Upper	*P* Value*
OC	−0.036	0.964	0.010	0.946	0.984	3 × 10^−4^
LGN	−0.017	0.983	0.010	0.964	1.003	0.093
V1	0.017	1.017	0.010	0.997	1.038	0.085
V2	0.009	1.009	0.010	0.990	1.029	0.352
V5	0.008	1.008	0.010	0.988	1.028	0.423

Regions analyzed include the OC, LGN, primary visual cortex (V1), and secondary visual cortex (V2 and V5). CI, confidence interval.

* A *P* value <0.005 is considered statistically significant after Bonferroni correction for 10 tests.

### Causal Inference

The MR analyses revealed no statistically significant causal association between POAG and the volumes of visual pathway regions in either direction. MR results using the inverse variance weighted method are presented in Tables 3 and 4, while MR sensitivity analysis results and plots are available in Supplementary Tables S2 to S11 and Supplementary Figures S1 and S2, respectively.

**Table 3.** Mendelian Randomization Results Using the Inverse Variance Weighted Method to Test Causality Between POAG as an Outcome and Volumes of Visual Pathway Regions as Exposures

Exposure	Outcome	**β**	SE	*P* Value^*^
OC	POAG	−0.077	0.093	0.409
LGN	POAG	−0.055	0.100	0.578
V1	POAG	0.001	0.033	0.981
V2	POAG	−0.063	0.041	0.123
V5	POAG	0.081	0.063	0.201

Regions analyzed include the OC, LGN, primary visual cortex (V1), and secondary visual cortex (V2 and V5).

* A *P* value <0.005 is considered statistically significant after Bonferroni correction for 10 tests.

**Table 4.** Mendelian Randomization Results Using the Inverse Variance Weighted Method to Test Causality Between POAG as an Exposure and Volumes of Visual Pathway Regions as Outcomes

Exposure	Outcome	**β**	SE	*P* Value^*^
POAG	OC	−0.013	0.007	0.063
POAG	LGN	−0.014	0.006	0.015
POAG	V1	−0.002	0.006	0.783
POAG	V2	−0.009	0.006	0.136
POAG	V5	−0.003	0.006	0.592

Regions analyzed include the OC, LGN, primary visual cortex (V1), and secondary visual cortex (V2 and V5).

* A *P* value <0.005 is considered statistically significant after Bonferroni correction for 10 tests.

### Colocalization

The GWAS-PW analysis identified eight shared genomic regions with causal variants common to both POAG and OC, with the region on chromosome 9, containing the CDKN2 gene family, among the identified regions. This gene family has an established association with POAG in previous studies.^6^ In addition, six regions were shared with V2, five regions with V1, and one region each with V5 and LGN. The details of the significant shared regions are available in Supplementary Table S12.

### SMR

The SMR analyses, using eQTLGen, revealed five potential causal genes shared between POAG and visual pathway regions that met the significance criteria (i.e., SMR *P* < 0.05/number of genes in the GWAS-PW identified regions and HEIDI test *P* > 0.05) and located within regions highlighted by GWAS-PW. In particular, the analyses identified a few significant genes shared with V1, including *PHETA1*, a gene involved in endosomal trafficking that may play a role in regulating membrane dynamics by interacting with the inositol polyphosphate 5-phosphatase *OCRL-1* enzyme,^41^ and *MAPKAPK5-AS1*, a long noncoding RNA (lncRNA) gene that has a role in cancer and rheumatoid arthritis.^42^^–^^44^ Furthermore, the *EEF1AKMT2* gene was found to be shared between POAG and V2, which is involved in protein methylation.^45^^,^^46^ Table 5 presents the full list of the shared potential causal genes. The identified potential causal genes were not targets of existing drugs in the Open Targets platform. Also, the SMR analyses using retinal tissues have not identified any significant results.

**Table 5.** Significant Genes Shared Between POAG and Visual Pathway Regions Identified by SMR Analysis (SMR *P* Value Survived Multiple Testing and HEIDI Test *P* > 0.05) and Located Within GWAS-PW Highlighted Regions

			Trait			POAG		
Trait	Gene	Location	SMR Estimate (SE)	SMR *P* Value^*^	HEIDI *P* Value	SMR Estimate (SE)	SMR *P* Value^*^	HEIDI *P* Value
V1	ENSG00000198324 (*PHETA1*, *FAM109A*)	Chr12, chunk 1245	0.174 (0.049)	2 × 10^−4^	0.810	0.197 (0.069)	3 × 10^−4^	0.337
V1	ENSG00000234608 (*MAPKAPK5-AS1*)	Chr12, chunk 1245	0.061 (0.018)	2 × 10^−7^	0.069	0.069 (0.025)	2 × 10^−7^	0.061
V1	ENSG00000204852 (*TCTN1*)	Chr12, chunk 1245	−0.020 (0.055)	7 × 10^−4^	0.324	0.102 (0.078)	6 × 10^−6^	0.056
V2	ENSG00000203791 (*EEF1AKMT2, METTL10*)	Chr10, chunk 1087	−0.125 (0.027)	6 × 10^−5^	0.157	−0.222 (0.043)	3 × 10^−5^	0.586
V2	ENSG00000257877	Chr12, chunk 1245	0.028 (0.017)	2 × 10^−4^	0.080	0.081 (0.028)	3 × 10^−5^	0.280

* A *P* value <0.05/the number of genes present in the regions of interest identified by GWAS-PW is considered statistically significant after Bonferroni correction. In particular, adjustments were conducted for 121 genes in OC, 5 in LGN, 62 in V1, 61 in V2, and 15 in V5.

### Expression of Prioritized Genes in the Occipital Cortex and Retinal Tissues

We observed that the putative causal genes were expressed in the occipital cortex and retinal tissues (Supplementary Table S13). Given that *MAPKAPK5-AS1* is an lncRNA gene, we instead examined the expression of the protein-coding *MAPKAPK5* as a proxy using the HPA platform. The novel transcript ENSG00000257877 lacked expression data on the platform. The *TCTN1* and *MAPKAPK5* genes were highly expressed in the occipital cortex compared to the other genes (nTPM = 23.3 and 22.1, respectively). In addition, the *MAPKAPK5* gene was highly expressed in the retina. FANTOM5 CAGE data revealed variable promoter activities across the prioritized genes in the occipital cortex and retina, with the expression level ranging from 3.9 to 56.2 in the occipital cortex and 3.8 to 62.5 in the retina, indicating differential gene regulation and expression in these tissues.

### Expression of Prioritized Genes in Single-Cell Transcriptomics

The *EEF1AKMT2* and *MAPKAPK5-AS1* genes were expressed in RGCs, which are the primary cell types that degenerate in glaucoma. The *TCTN1* gene was expressed in a subset of rods and cones, which are the primary photoreceptor cells in the retina. In addition, *MAPKAPK5-AS1*, *EEF1AKMT2*, and *TCTN1* were expressed in the trabecular meshwork and ciliary body cells, which are relevant in IOP regulation (Supplementary Fig. S3). For the visual cortex, *MAPKAPK5-AS1*, *EEF1AKMT2*, and *TCTN1* genes were expressed in excitatory and inhibitory neuronal cells. However, none of these genes were expressed in nonneuronal cells (Supplementary Fig. S4).

## Discussion

To date, no studies have investigated the genetic overlap between POAG and the regions of the visual pathway across the genome. Here, we explored the shared genetic architecture between five brain regions and POAG. In particular, we examined the genetic correlation, PRS, causal association, colocalization, and shared potential causal genes between POAG and the volumes of those brain areas in the shared genomic regions. The results revealed significant genetic correlation and PRS association between OC and POAG, while the other visual regions did not show significant findings. However, GWAS-PW identified a few shared genetic regions between POAG and the five visual regions, and SMR analyses identified five shared candidate causal genes.

POAG primarily affects the optic nerve, and the current literature suggests that it may also impact the visual pathway regions in the brain.^8^^–^^14^ In this study, we show that despite no statistically significant causal association being observed, the effect sizes were consistent in their direction. However, the results remain inconclusive, likely because the power to detect causality for small to modest effect sizes was limited due to the small sample size.

The genetic correlation and PRS results indicate that OC is biologically related to POAG. That could be due to the fact that OC is an extension of the optic nerve that is damaged during POAG-related neuropathy. The direction of the association observed in our analyses aligns with the findings from observational studies, which showed that individuals with POAG have decreased OC size, indicating an inverse relationship between both traits.^11^^–^^13^

The segment on chromosome 12, ranging from positions 110,336,875 to 113,261,782, includes four potential causal genes. One gene, *PHETA1*, is associated with the cup-to-disc ratio,^47^ a trait indicating optic nerve degeneration and closely related to POAG. Another gene is *MAPKAPK5-AS1*, which is an lncRNA gene (i.e., lncRNAs are important regulators of gene expression and cellular processes) that has been shown to promote diseases by regulating the adjacent gene *MAPKAPK5*.^48^ The *MAPKAPK5* gene has a role in neuronal plasticity and cell cycle.^49^ It has also been associated with Alzheimer's disease pathogenesis, and it has been presented as a potential biomarker for cognitive decline.^50^^,^^51^ Also, both genes were previously associated with POAG^6^; however, further research in the specific roles of *PHETA1* and *MAPKAPK5-AS1* genes in POAG could facilitate the development of novel therapeutic molecules targeting these genes.

The region on chromosome 10 contains one candidate causal gene. The *EEF1AKMT2* gene encodes a protein that functions as protein-lysine N-methyltransferase, an enzyme that regulates gene expression and cellular processes through posttranslational modification.^45^^,^^46^ The regulation of protein synthesis is vital for all cells’ health and survival, including neurons. This gene was associated with the hippocampus volume, a region implicated in brain diseases such as Alzheimer's disease and schizophrenia.^52^^,^^53^ Given that this gene was also mapped for POAG,^6^ this observation highlights the need for further investigation into the role of the *EEF1AKMT2* gene in glaucoma etiology and as a treatment target.

This study has several limitations that should be acknowledged. For instance, we did not investigate other glaucoma subtypes since most glaucoma cases in Europeans are POAG, and previous observational studies have concentrated on this subtype. Additionally, the analyses were focused on POAG and did not include POAG subtypes such as NTG, as the currently available summary statistics lack sufficient power to draw meaningful conclusions in this study for NTG. Future studies with larger data sets will be needed to investigate the potential associations between NTG and visual pathway regions in the brain. Moreover, this study is limited to the European population, which may lack the genetic diversity present in other ethnic groups. As a result, the findings cannot be generalized to other populations. In addition, the power of MR analysis to detect small to modest causal relationships may have been limited by the sample size of POAG and the brain regions. Similarly, the sample size of the volumes of visual pathway regions is relatively small, which potentially limits the statistical power to detect genetic variants. Larger cohorts would provide better power to identify additional overlapping genes and more robust biological pathways. While we identified certain genes associated with POAG and visual pathways, functional validation of these genes was not performed. Without experimental validation, the biological relevance of these associations remains uncertain. Further studies are needed to confirm the functional impact of the identified genes.

Our findings suggest a genetic overlap between POAG and the volumes of visual pathway regions in the brain, including shared candidate causal genes. Further investigations are required to elucidate the specific role of the shared genes in the etiology of POAG and to assess their potential as neuroprotective drug targets.

## Supplementary Material

Supplement 1

Supplement 2
