# Supplementary material for: Exploring the Shared Genetic Architectures Between Primary Open-Angle Glaucoma and Visual Pathway Regions in the Brain
Source: Invest Ophthalmol Vis Sci. 2025 Dec 2;66(15):11. doi: 10.1167/iovs.66.15.11 (PMC12697700; doi:10.1167/iovs.66.15.11)

**Supplementary Figures**

**Exploring the Shared Genetic Architectures between Primary Open-Angle Glaucoma and Visual Pathway Regions in the Brain**

**Supplementary Figure 1.** Mendelian Randomization analysis evaluating the causal effect of the volume of each visual pathway region (i.e., exposure) on POAG (i.e., outcome). Each point represents a genetic variant as instrumental variable for the exposure, with the X-axis showing the effect on the exposure and the Y-axis showing the effect on the outcome. The lines indicate the estimated causal effect derived from the following methods: inverse variance weighted, MR-Egger, simple mode, both weighted median and mode.


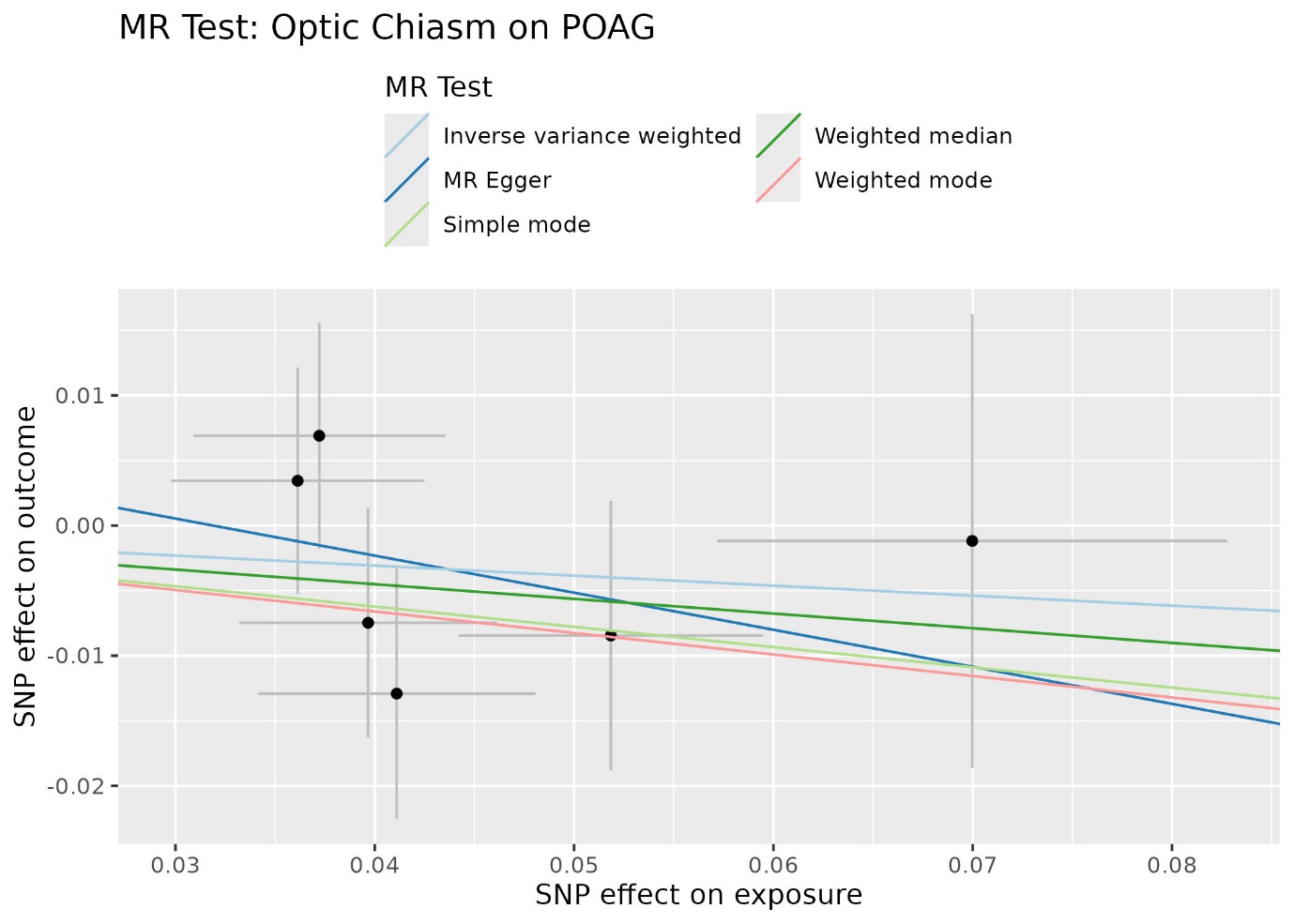


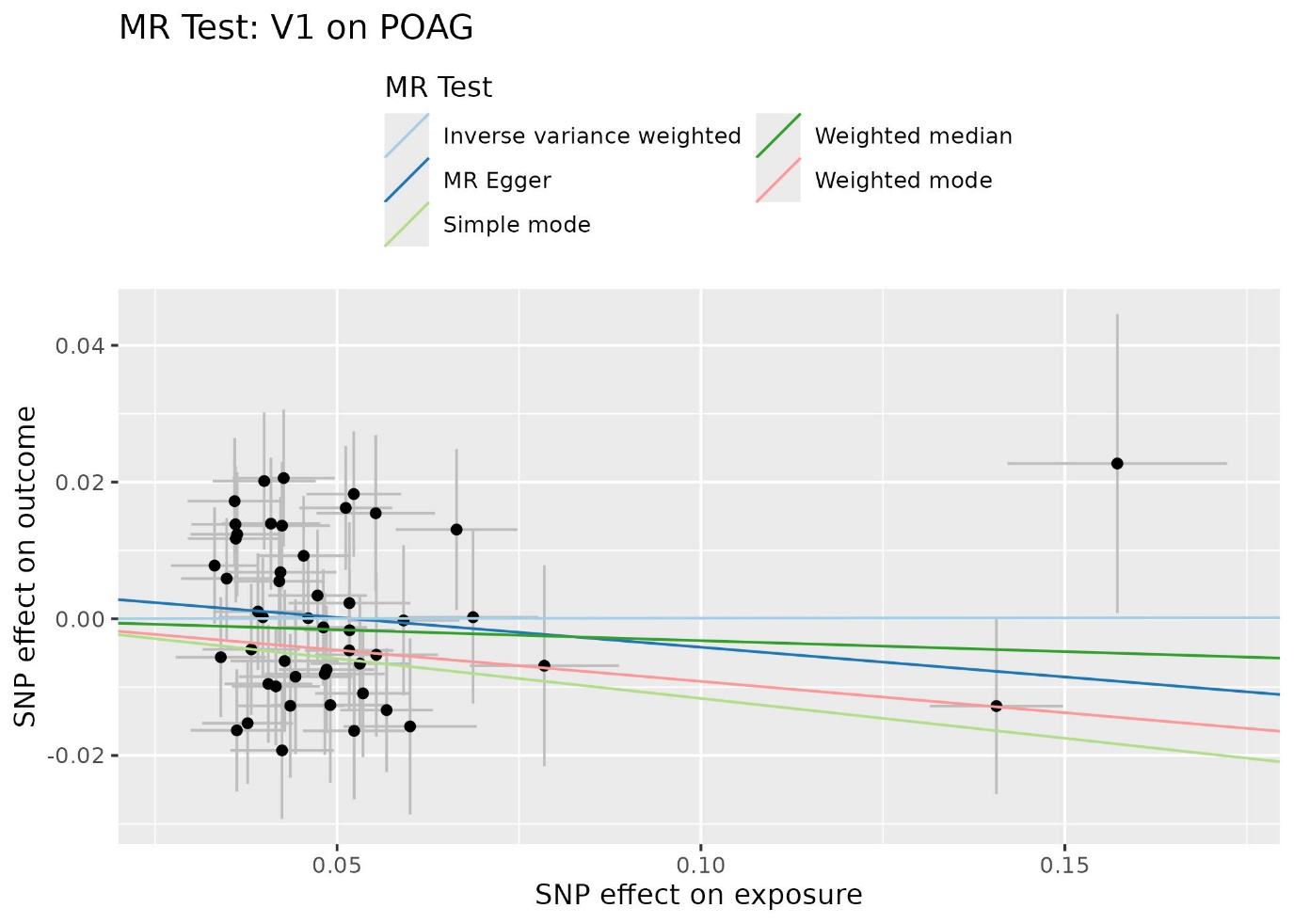

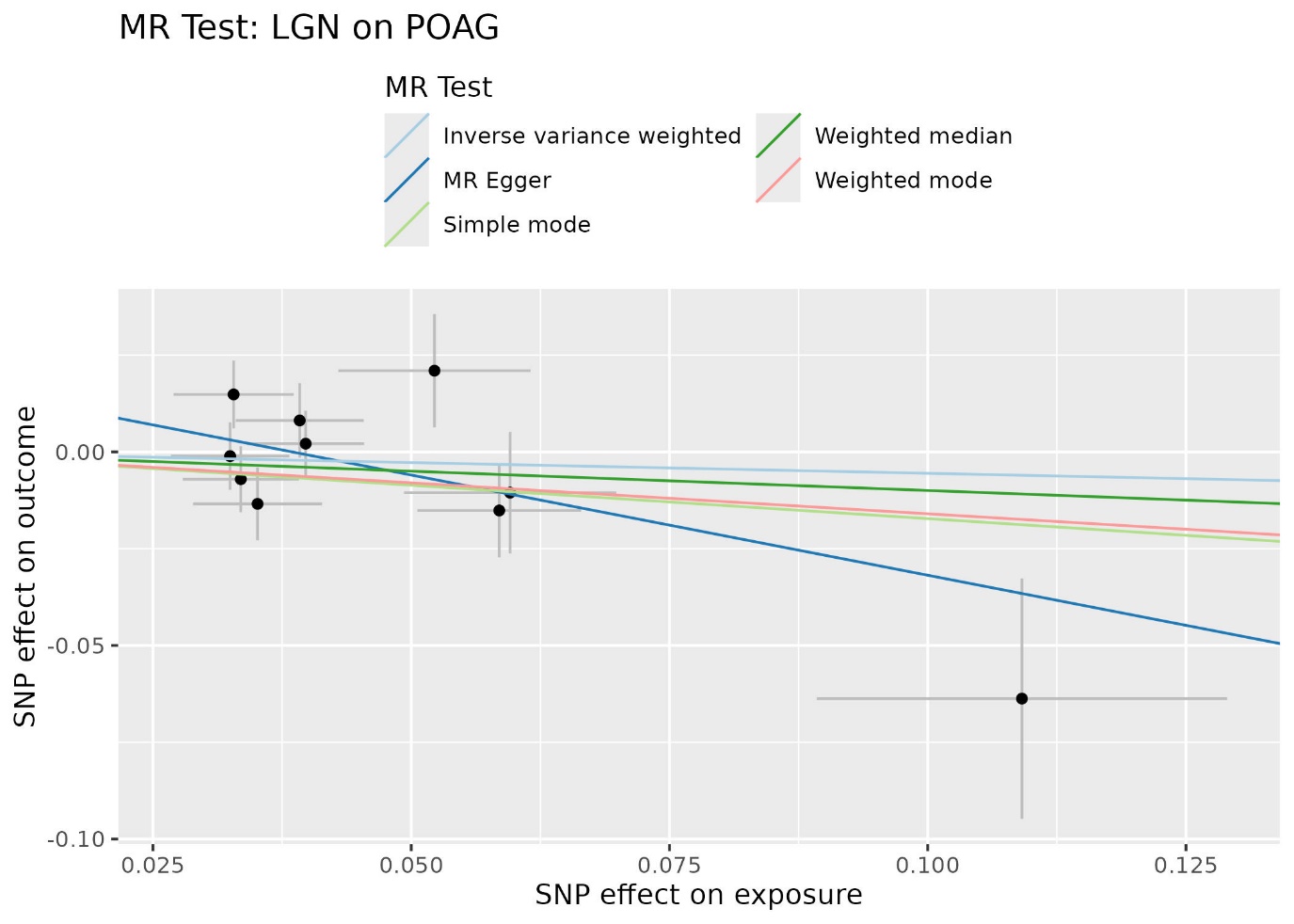


## **
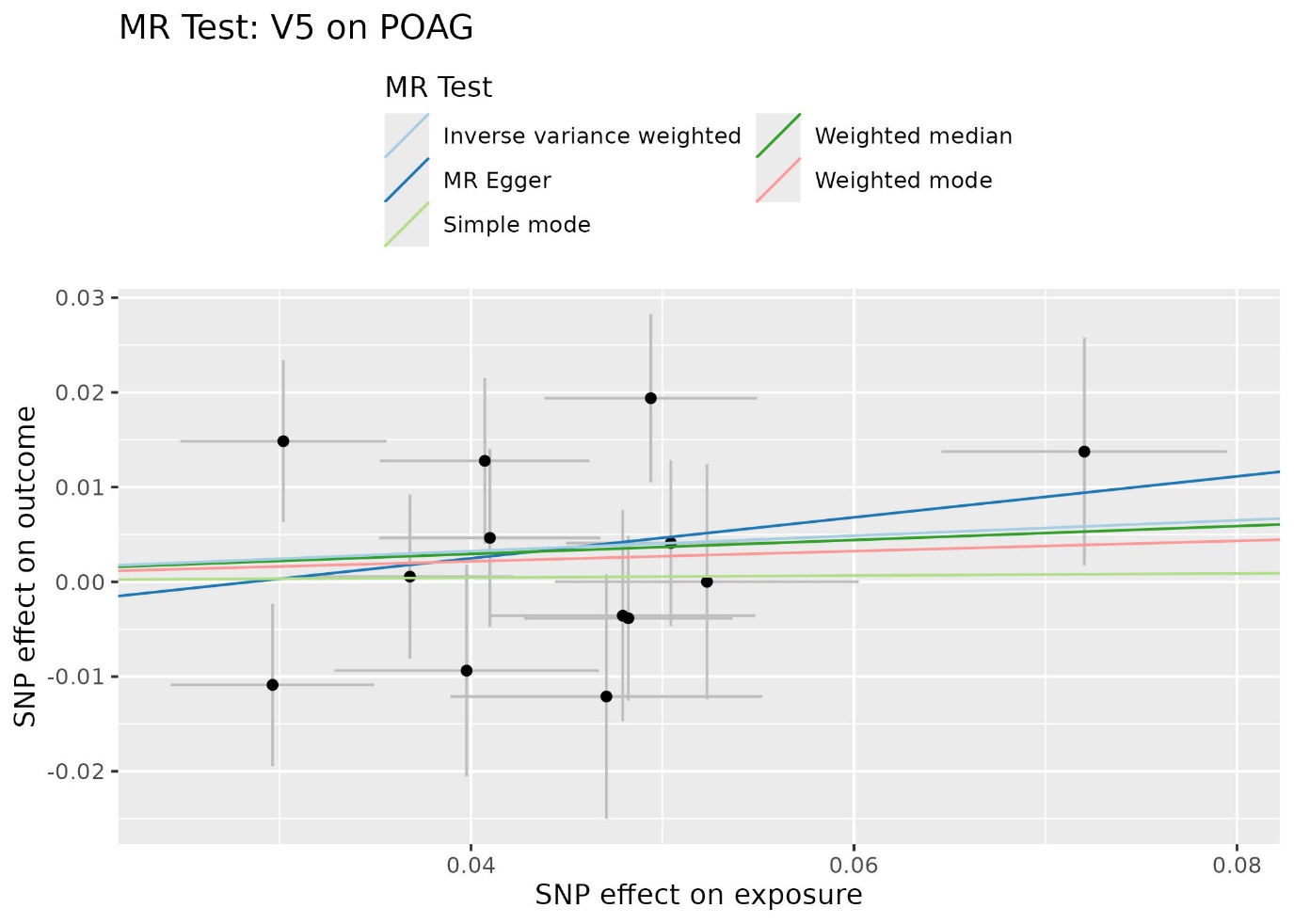

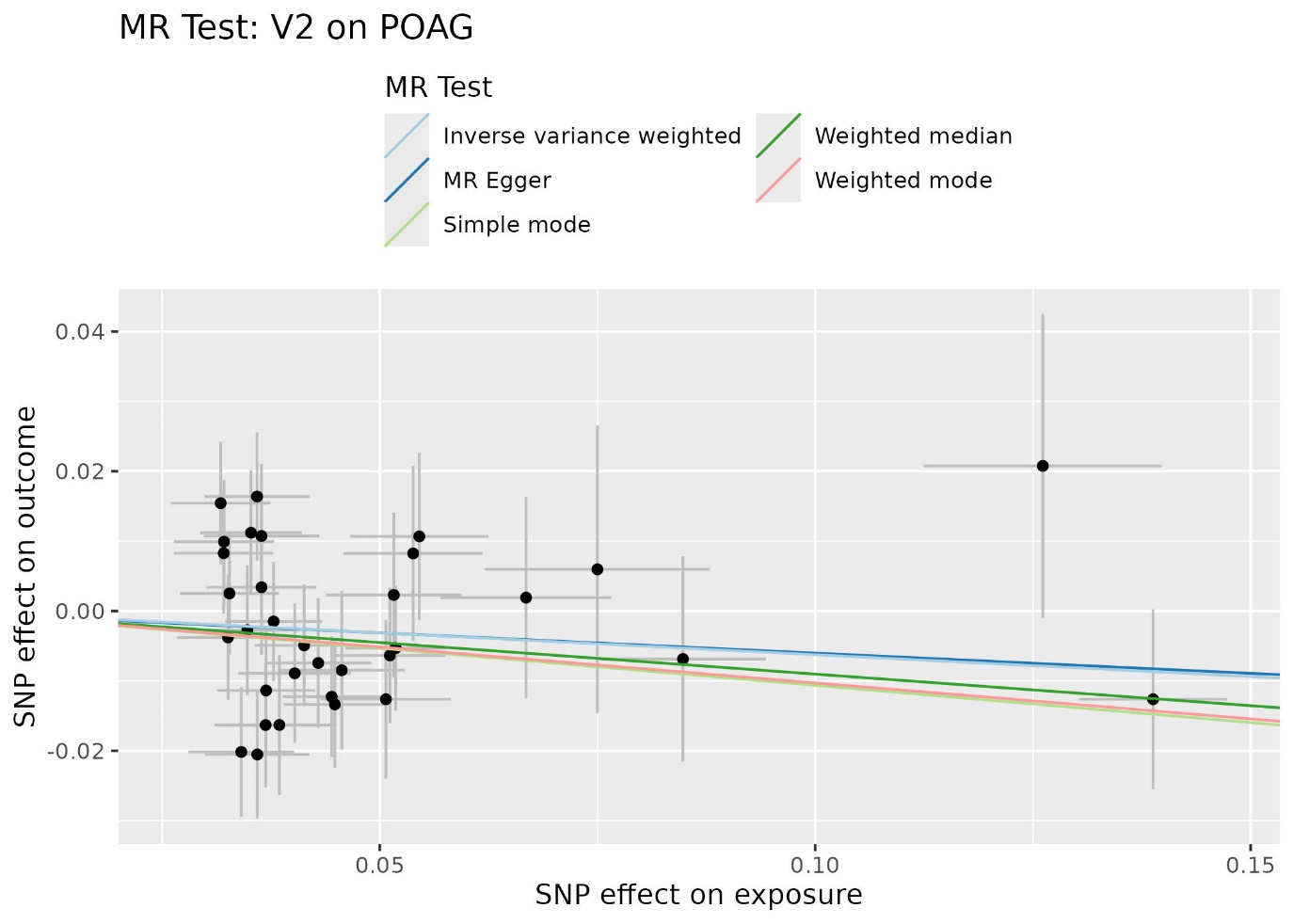
**

**Supplementary Figure 2.** Mendelian Randomization analysis evaluating the causal effect of POAG (i.e., exposure) on the volume of each visual pathway region (i.e., outcome). Each point represents a genetic variant as instrumental variable for the exposure, with the X-axis showing the effect on the exposure and the Y-axis showing the effect on the outcome. The lines indicate the estimated causal effect derived from the following methods: inverse variance weighted, MR-Egger, simple mode, both weighted median and mode.


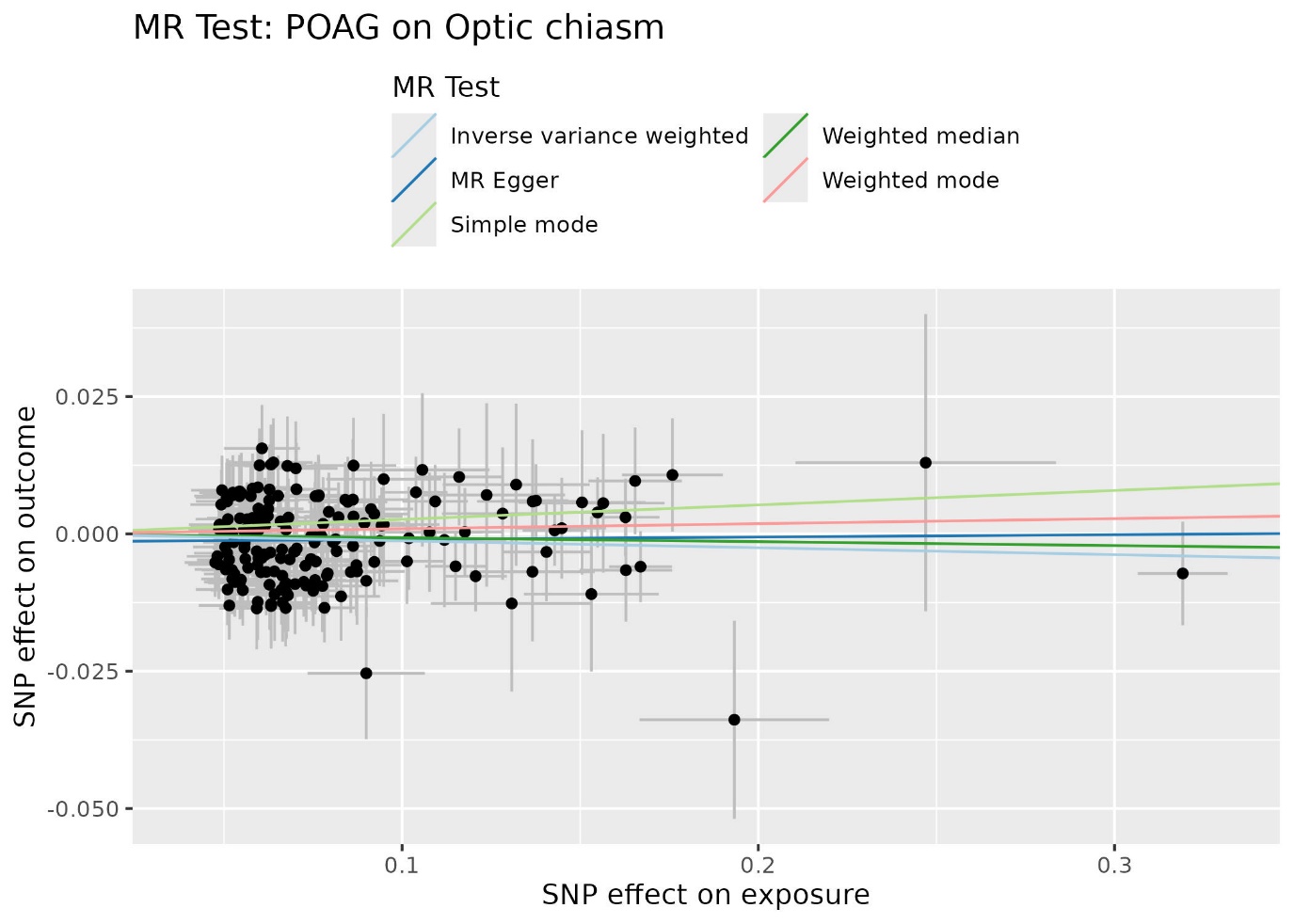


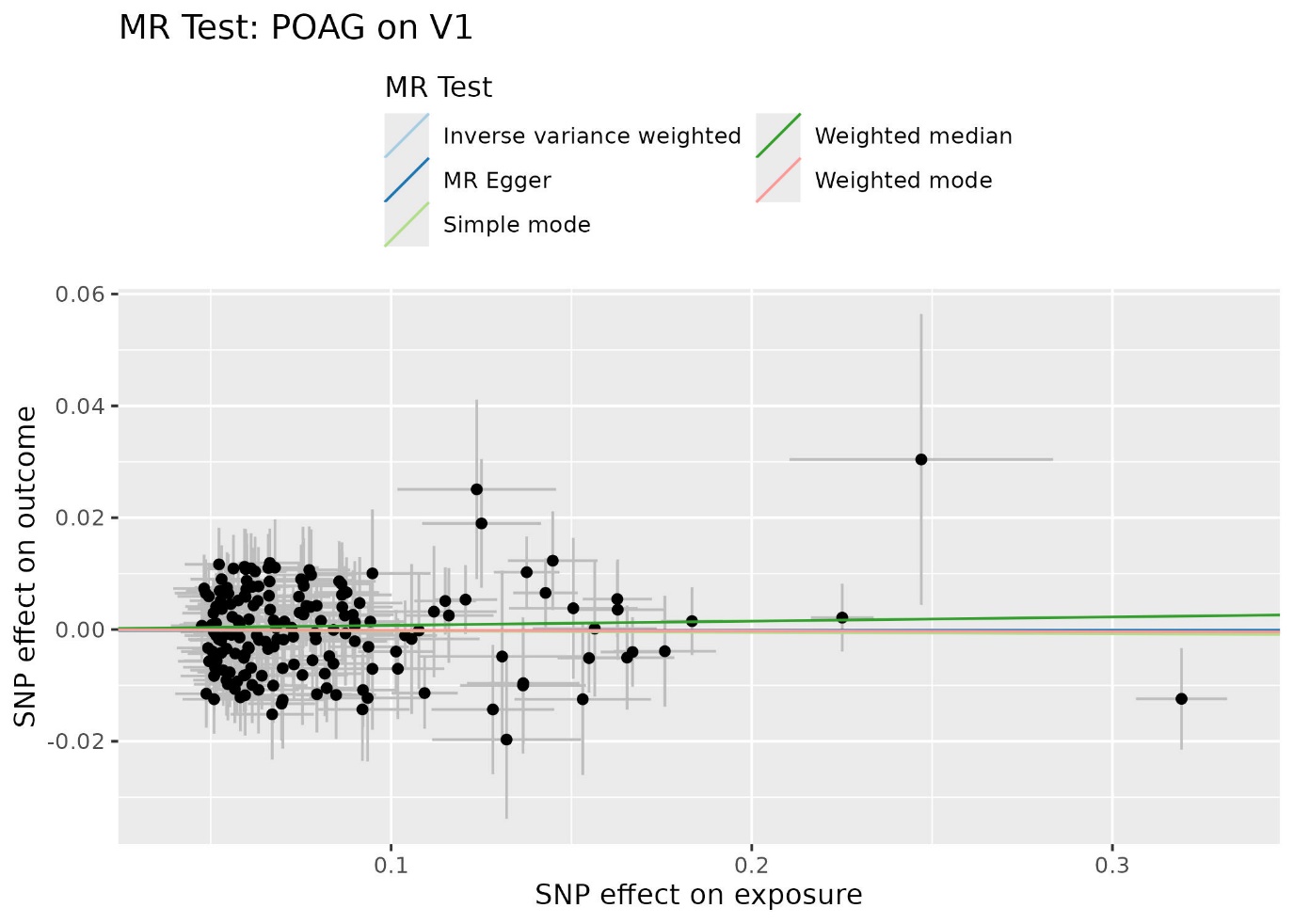

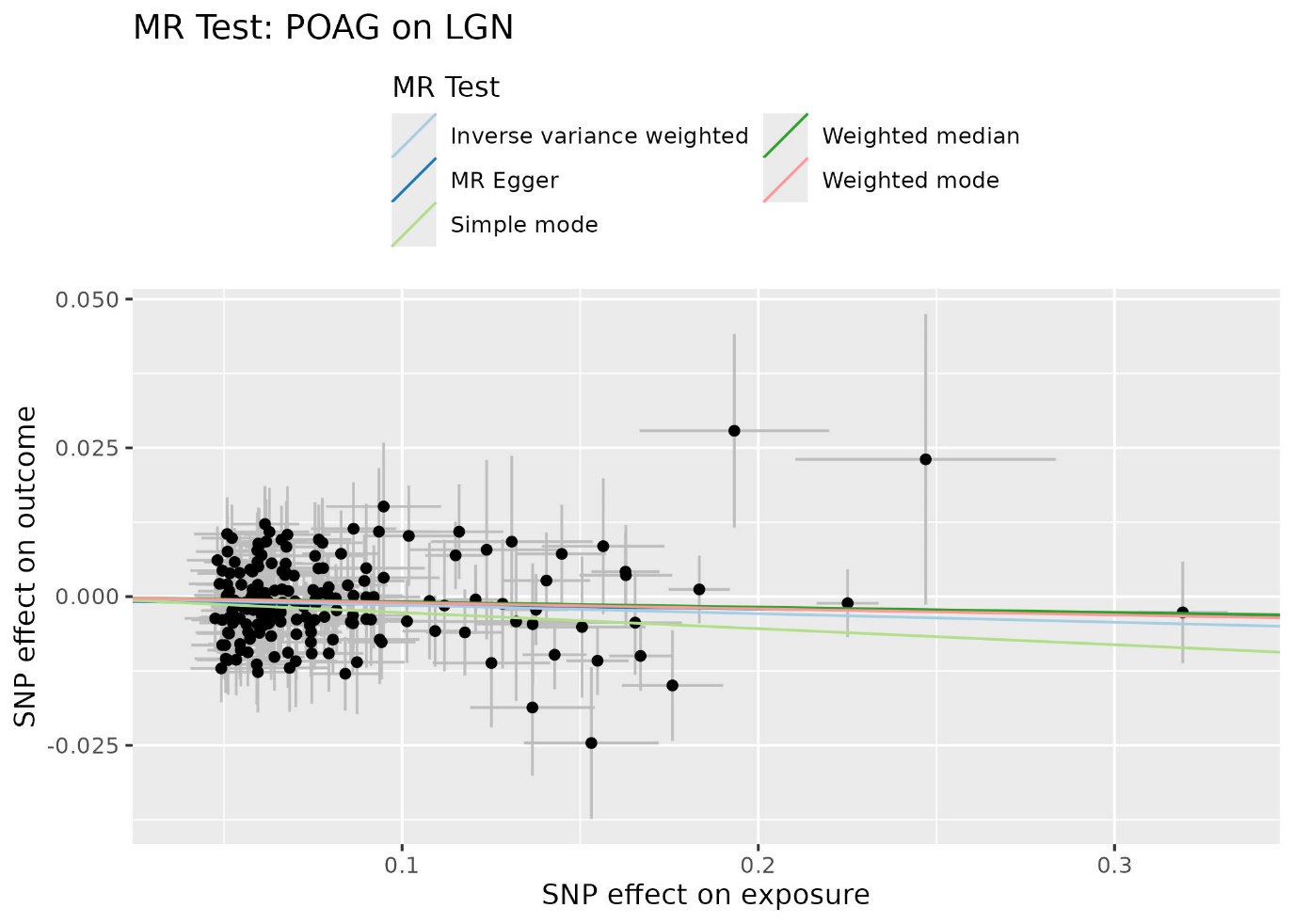


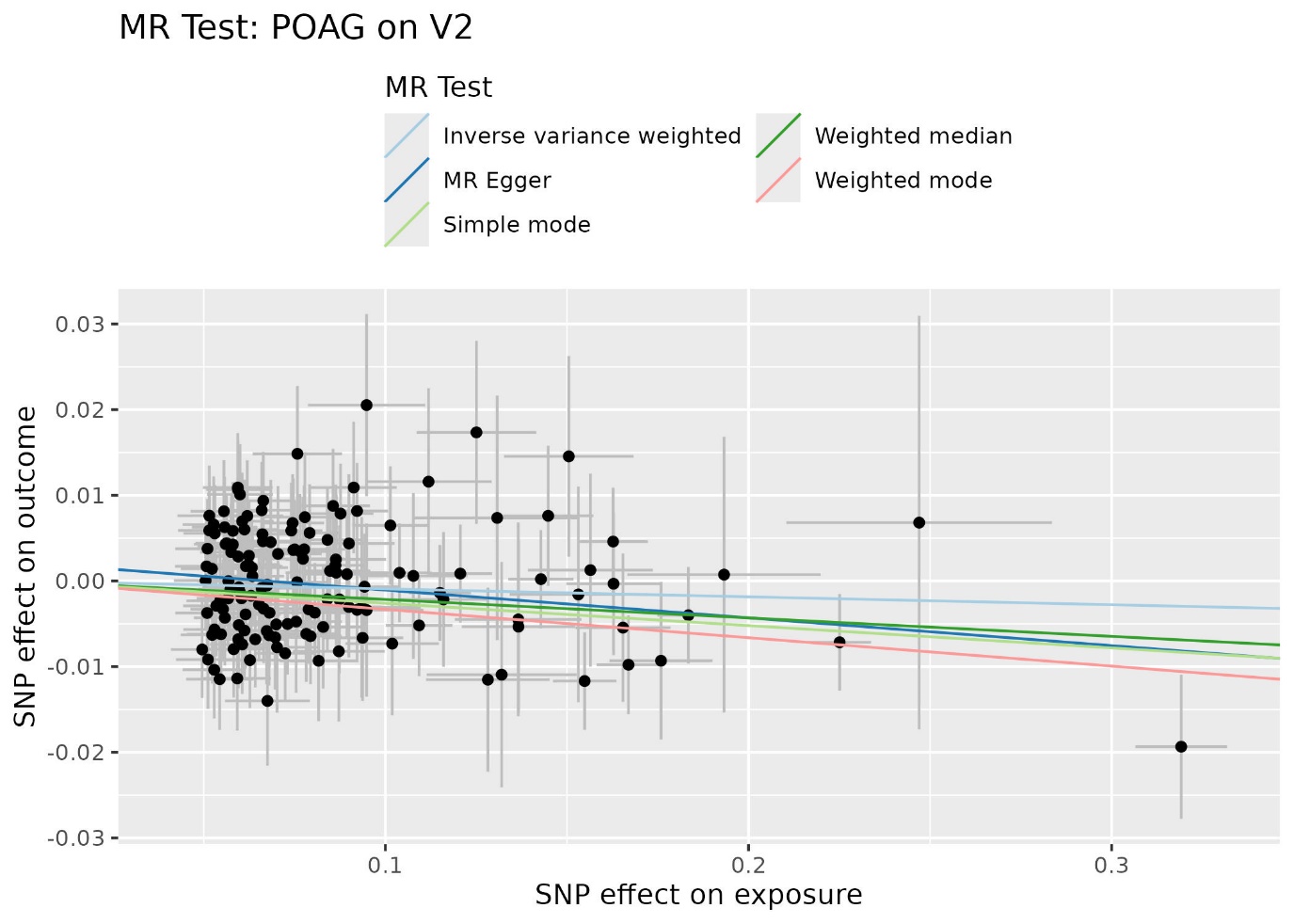


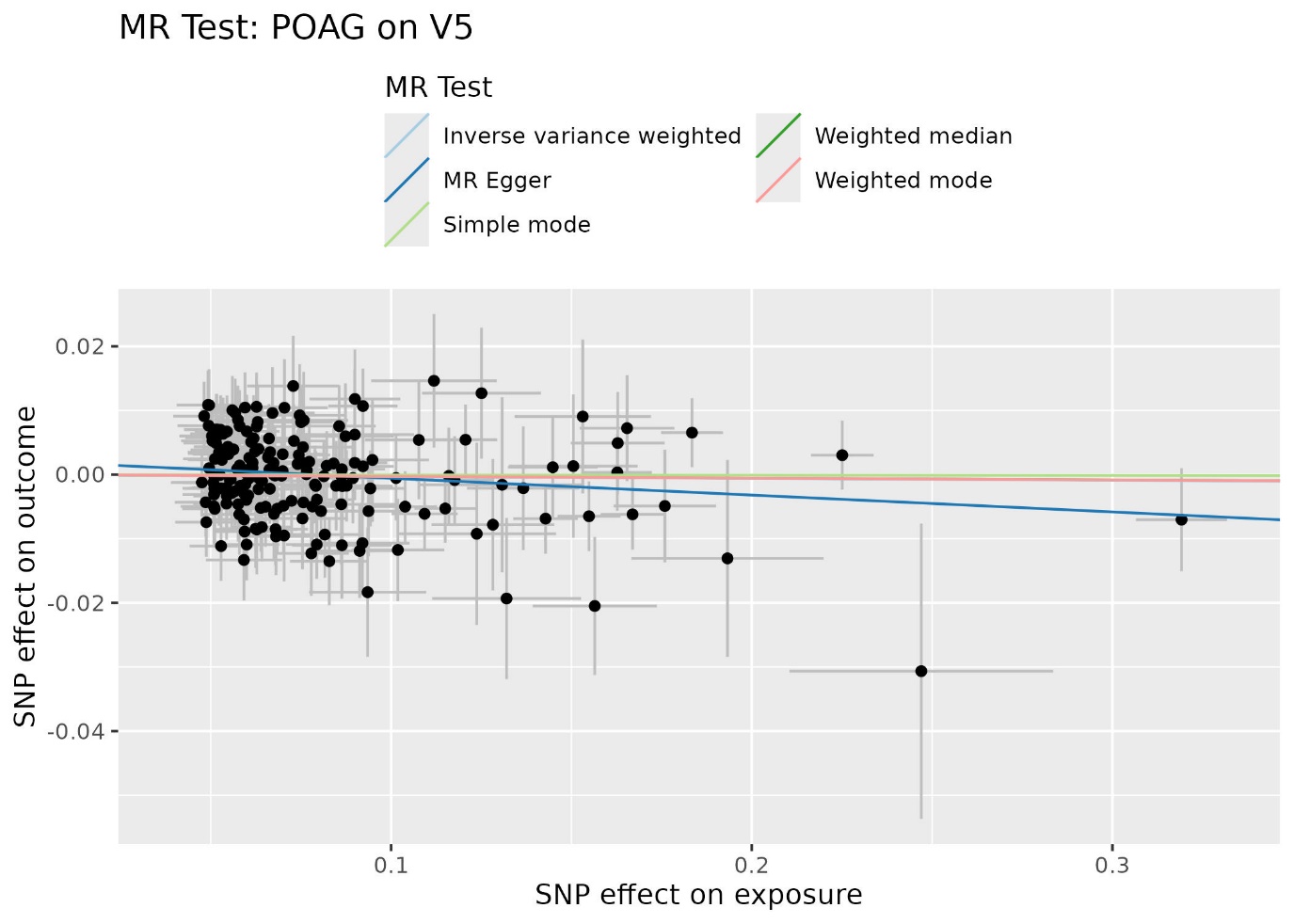


**Supplementary Figure 3.** Dot plots showing log-normalized expression of the putative causal genes, including *PHETA1* (ENSG00000198324), *MAPKAPK5-AS1* (ENSG00000234608), *TCTN1* (ENSG00000204852), and *EEF1AKMT2* (ENSG00000203791) across different cell types in (a) the retina and (b) trabecular meshwork and ciliary body. The size of each dot represents the percentage of cells within a given cell type that express the gene, while the color intensity indicates the average expression level among expressing cells. Gene symbols are shown on the x-axis and cell types on the y-axis.

1. **Retina
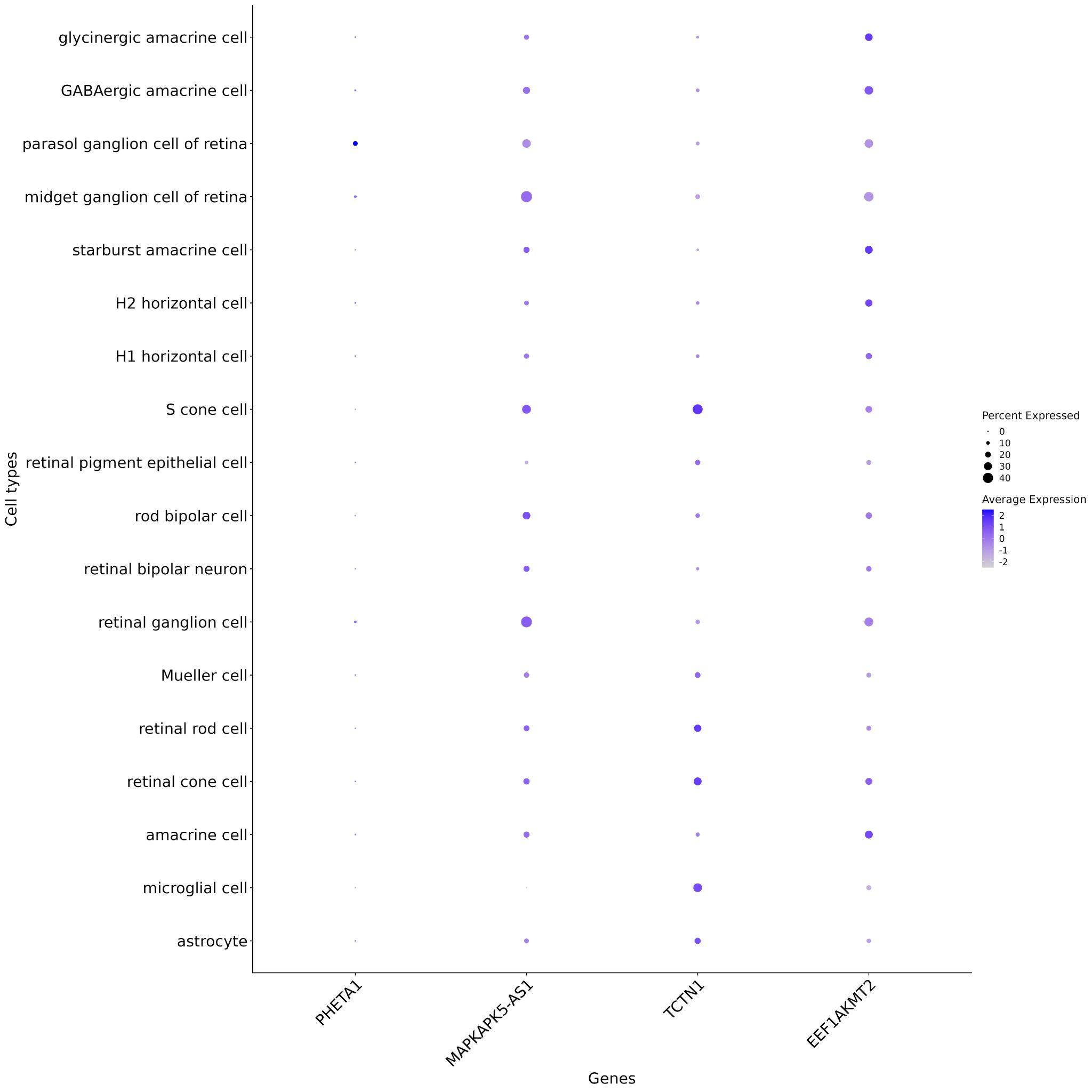
**
2. **Trabecular meshwork and ciliary body
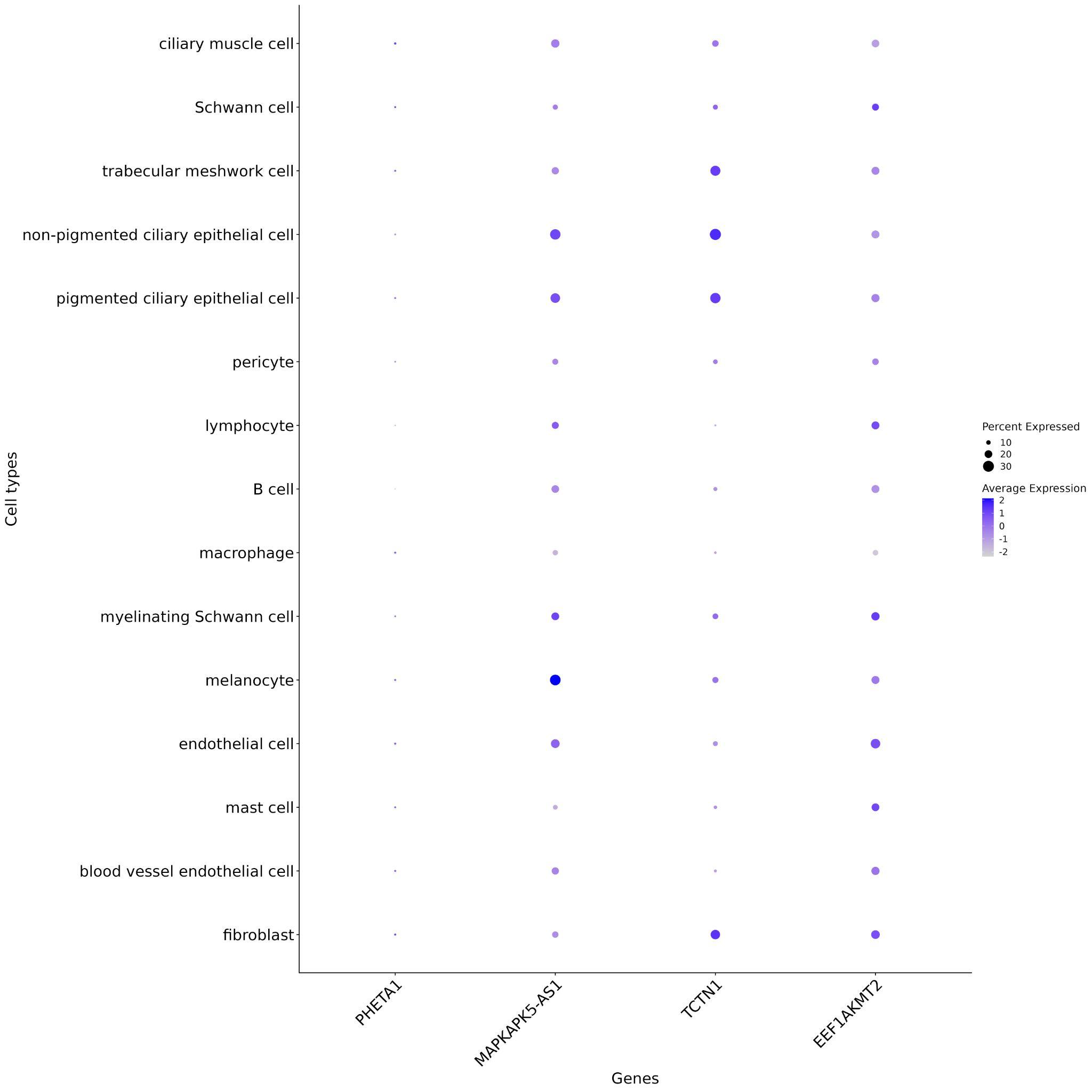
**

**Supplementary Figure 4.** Dot plot showing log-normalized expression of the putative causal genes, including *PHETA1* (ENSG00000198324), *MAPKAPK5-AS1* (ENSG00000234608), *TCTN1* (ENSG00000204852), and *EEF1AKMT2* (ENSG00000203791) across different cell types in the visual cortex. The size of each dot represents the percentage of cells within a given cell type that express the gene, while the color intensity indicates the average expression level among expressing cells. Gene symbols are shown on the x-axis and cell types on the y-axis.


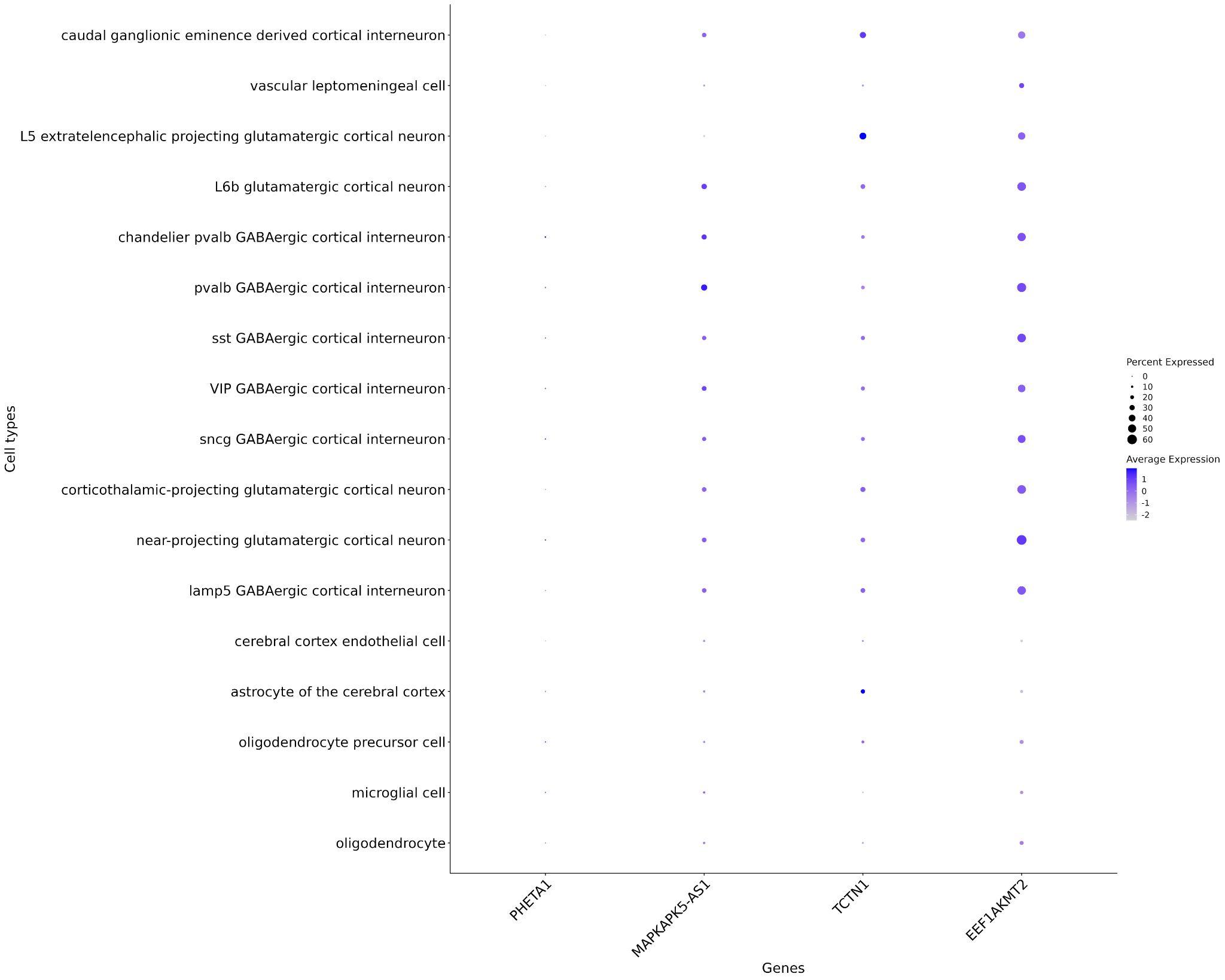

Supplement: Supplement 1 [file iovs-66-15-11_s001.docx]
